# Changes Induced by Early Hand-Arm Bimanual Intensive Therapy Including Lower Extremities in Young Children With Unilateral Cerebral Palsy: A Randomized Clinical Trial

**DOI:** 10.1001/jamapediatrics.2023.4809

**Published:** 2023-11-06

**Authors:** Rodrigo Araneda, Daniela Ebner-Karestinos, Julie Paradis, Anne Klöcker, Geoffroy Saussez, Josselin Demas, Rodolphe Bailly, Sandra Bouvier, Astrid Carton de Tournai, Enimie Herman, Aghiles Souki, Grégoire Le Gal, Emmanuel Nowak, Stephane V. Sizonenko, Christopher J. Newman, Mickael Dinomais, Inmaculada Riquelme, Andrea Guzzetta, Sylvain Brochard, Yannick Bleyenheuft

**Affiliations:** 1 Institute of Neuroscience, Université catholique de Louvain, Brussels, Belgium; 2 Exercise and Rehabilitation Science Institute, Faculty of Rehabilitation Science, Universidad Andres Bello, Santiago, Chile; 3 Department of Developmental Neuroscience, IRCCS Fondazione Stella Maris, Pisa, Italy; 4 Haute Ecole Léonard de Vinci, Parnasse-ISEI, Brussels, Belgium; 5 Forme et Fonctionnement Humain Unit, Department of Motor Sciences, CeREF - Haute Ecole Louvain en Hainaut, Belgium; 6 Université d’Angers, Laboratoire Angevin de Recherche en Ingénierie des Systèmes (LARIS) – EA7315 F-49000 France; 7 Instituts de formation du Centre Hospitalier de Laval, Laval, France; 8 INSERM UMR 1101, LaTIM, Brest, France; 9 Pediatric Rehabilitation Department, Fondation Ildys, Brest, France; 10 Western Brittany University, Brest, France; 11 University Hospital of Brest, Brest, France; 12 INSERM CIC 1412, Brest, France; 13 Division of Child Development and Growth, Department of Pediatrics, University of Geneva, Geneva, Switzerland; 14 Paediatric Neurology and Neurorehabilitation Unit, Lausanne University Hospital, University of Lausanne, Lausanne, Switzerland; 15 CHU Angers, Département de Médecine Physique et de Réadaptions, CHU Angers-Capucins, F- 49933, France; 16 Department of Nursing and Physiotherapy and Research Institute on Health Sciences (UINICS-Idisba), University of the Balearic Islands, Palma de Mallorca, Spain; 17 Department of Clinical and Experimental Medicine, University of Pisa, Pisa, Italy

## Abstract

**Importance:**

Intensive interventions are provided to young children with unilateral cerebral palsy (UCP), classically focused on the upper extremity despite the frequent impairment of gross motor function. Hand-Arm Bimanual Intensive Therapy Including Lower Extremities (HABIT-ILE) effectively improves manual dexterity and gross motor function in school-aged children.

**Objective:**

To verify if HABIT-ILE would improve manual abilities in young children with UCP more than usual motor activity.

**Design, Setting, and Participants:**

This prospective randomized clinical trial (November 2018 to December 2021), including 2 parallel groups and a 1:1 allocation, recruitment took place at European university hospitals, cerebral palsy specialized centers, and spontaneous applications at 3 sites: Brussels, Belgium; Brest, France; and Pisa, Italy. Matched (age at inclusion, lesion type, cause of cerebral palsy, and affected side) pairs randomization was performed. Young children were assessed at baseline (T0), 2 weeks after baseline (T1), and 3 months after baseline (T2). Health care professionals and assessors of main outcomes were blinded to group allocation. At least 23 young children (in each group) aged 12 to 59 months with spastic/dyskinetic UCP and able to follow instructions were needed. Exclusion criteria included uncontrolled seizures, scheduled botulinum toxin injections, orthopedic surgery scheduled during the 6 months before or during the study period, severe visual/cognitive impairments, or contraindications to magnetic resonance imaging.

**Interventions:**

Two weeks of usual motor activity including usual rehabilitation (control group) vs 2 weeks (50 hours) of HABIT-ILE (HABIT-ILE group).

**Main Outcomes and Measures:**

Primary outcome: Assisting Hand Assessment (AHA); secondary outcomes: Gross Motor Function Measure-66 (GMFM-66), Pediatric Evaluation of Disability Inventory-Computer Adaptive Test (PEDI-CAT), and Canadian Occupational Performance Measure (COPM).

**Results:**

Of 50 recruited young children (26 girls [52%], median age; 35.3 months for HABIT-ILE group; median age, 32.8 months for control group), 49 were included in the final analyses. Change in AHA score from T0 to T2 was significantly greater in the HABIT-ILE group (adjusted mean score difference [MD], 5.19; 95% CI, 2.84-7.55; *P* < .001). Changes in GMFM-66 (MD, 4.72; 95% CI, 2.66-6.78), PEDI-CAT daily activities (MD, 1.40; 95% CI, 0.29-2.51), COPM performance (MD, 3.62; 95% CI, 2.91-4.32), and satisfaction (MD, 3.53; 95% CI, 2.70-4.36) scores were greater in the HABIT ILE group.

**Conclusions and Relevance:**

In this clinical trial, early HABIT-ILE was shown to be an effective treatment to improve motor performance in young children with UCP. Moreover, the improvements had an impact on daily life activities of these children.

**Trial registration:**

ClinicalTrials.gov Identifier: NCT04020354

## Key Points

**Question:**

What is the effect of early Hand-Arm Bimanual Intensive Therapy Including Lower Extremities (HABIT-ILE) intervention on bimanual performance vs usual, unstructured spontaneous motor activity in children between 1 and 4 years old with unilateral cerebral palsy after 3 months?

**Findings:**

This randomized clinical trial including 50 children found improvements in bimanual hand function scores that were significantly higher in the HABIT-ILE group than in the control group.

**Meaning:**

Early HABIT-ILE improved bimanual performance more than usual motor activity in young children with unilateral cerebral palsy.

## Teaser

This study attempts to verify if Hand-Arm Bimanual Intensive Therapy Including Lower Extremities would improve manual abilities in young children with unilateral cerebral palsy more than usual motor activity.

## Introduction

Cerebral palsy (CP) is the most common pediatric motor disorder, occurring in 2 to 2.5 per 1000 live births.^1,2^ The initial lesion or maldevelopment occurs before the age of 2 years and can affect different periods of brain growth. CP is caused by the ensuing atypical brain development, especially if the pathways for the control of skilled movements are compromised.^3,4,5,6,7^ The motor impairments caused by CP mostly affect the execution of daily life activities, primarily because of reduced manual abilities and gross motor function.^8^ These limitations may have a detrimental effect on the quality of life and participation of these children throughout their lives.^2^

Evidence supports the use of intensive goal-directed interventions based on motor skill learning to improve motor function and daily activities in school-aged children with CP, as compared with regular care.^9,10,11^ These interventions induce neuroplastic changes that result in improved function.^12,13^ However, the main activity-dependent brain reorganization produced by environmental experience occurs early in life. Providing interventions to children with CP during this window of opportunity could potentially maximize functional changes, positively impact the whole developmental curve, and minimize subsequent complications.^14,15,16,17^ However, few studies have evaluated the effectiveness of intensive rehabilitation in young children.^9,15,17^

Most intensive interventions investigated in young children with unilateral CP (UCP) involved constraint-induced movement therapy or bimanual training that only target the upper extremities, despite the frequent impairment of gross motor function, including the lower extremities and trunk.^18^ Hand-Arm Bimanual Intensive Therapy Including Lower Extremities (HABIT-ILE) is an intensive intervention that involves the practice of voluntary movement with many repetitions and progressive shaping in a child-friendly manner. In addition to stimulating bimanual coordination, HABIT-ILE includes continuous stimulation of the lower extremities and trunk.^19^ Recently, a single-group, self-controlled pilot study of the feasibility of HABIT-ILE in 10 preschool children with UCP found large differences in manual dexterity and gross motor function after 50 hours of therapy.^20^ It is now crucial to confirm these results in an adequately powered randomized clinical trial (RCT).

We aimed to evaluate the effect of HABIT-ILE against usual (spontaneous and unstructured) motor activity, including usual rehabilitation on bimanual performance at 3 months in children with UCP between 1 and 4 years old. We hypothesized that HABIT-ILE would improve bimanual ability and gross motor function more than usual, unstructured motor activity.

## Methods

Full ethical approval was obtained for this RCT in Belgium (B403201316810), France (29BRC19.0050/N2019-A01173–54), and Italy (244/2019). The parents of the young children included provided signed informed consent for their child’s participation. This study is part of a large multicenter European project including 2 RCTs: one for children with unilateral cerebral palsy and the second for children with bilateral cerebral palsy. This study is reported in accordance with the Consolidated Standards of Reporting Trials (CONSORT) reporting guidelines. The study protocol is available in Supplement 2.

### Study Population

**Figure 1.**
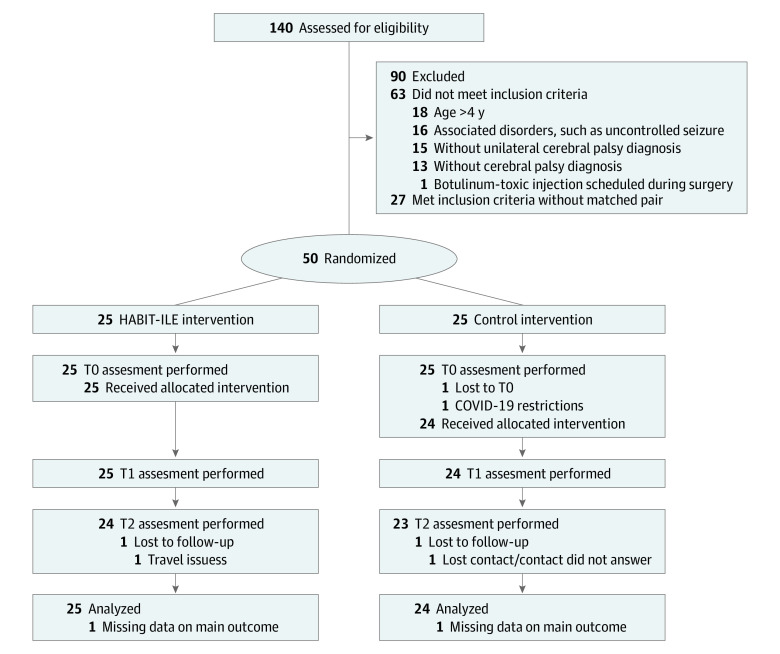
Flow Diagram of Participants HABIT-ILE indicates Hand-Arm Bimanual Intensive Therapy Including Lower Extremities; T0, first testing session (baseline); T1, second testing session 2 weeks after T0; T2, third testing session 3 months after T0.

Fifty young children (1 to 4 years old) were recruited from Belgian university hospital centers dedicated to the treatment of children with CP, from Brest University Hospital Centre in France, and from the IRCCS Fondazione Stella Maris in Pisa, Italy. Spontaneous applications from the parents of children were also considered. The inclusion criteria were young children with a diagnosis of unilateral spastic or dyskinetic CP, aged between 12 and 59 months (corrected age if preterm birth), and able to follow instructions. Half of the children recruited were aged 12 to 35 months, when descending motor pathways are likely to reorganize,^21,22^ and half were aged 36 to 59 months, when reorganization is less likely. Children were excluded if they had uncontrolled seizures, botulinum toxin injections, orthopedic surgery scheduled less than 6 months previously or scheduled during the study period, had severe visual or cognitive impairments that could interfere with the intervention and/or assessments, or had any contraindications to magnetic resonance imaging. Recruitment occurred across the 3 sites: Belgium (n = 18), France (n = 16), and Italy (n = 16) (Figure 1).

The young children were classified according to their manual performance using the Manual Ability Classification System for Children with CP aged 1 to 4 (Mini-MACS),^23^ and their gross motor function using the Gross Motor Function Classification System-Expanded and Revised (GMFM-66),^24^ and the corresponding modified versions for children younger than 2 years and those between 2 and 4 years old. Both systems involve a 5-level classification, with I indicating the highest motor ability (best performers) and V the lowest motor ability level.

### Study Design and Data Collection

This prospective multicenter RCT was a 2 parallel-group design with a 1:1 allocation ratio performed between November 2018 and December 2021. A matched-pairs randomization was performed at each site according to age at inclusion, lesion type (brain malformation/ periventricular white matter lesion/ gray matter lesion), and affected side (right/left). The young children were then randomly allocated to either the control or the treatment group using computer generated randomization. Health care professionals, as well as the assessors of the Assisting Hand Assessment (AHA), GMFM-66, and Melbourne Assessment 2 (MA2) were blinded to group allocation. The RCT compared the effect of 50 hours over 2 weeks of early HABIT-ILE with spontaneous, unstructured motor activity with an estimated activity time of around 50 hours over 2 weeks. Assessments were performed at 3 time points: baseline (T0), 2 weeks after baseline (T1), and 3 months after baseline (T2).

### Assessments

#### Primary Outcomes

The primary outcome was the between-group difference in score change (T2-T0) at 3 months in the AHA^25^ (AHA, children older than 18 months) or the version for children younger than 18 months (Mini-AHA^26^). This tool evaluates how children use their more affected hand to assist the less affected hand in bimanual activities. Scores are transformed into linear measures on a scale from 0 to 100 using a Rasch model (logit based AHA-unit). The session was video recorded for subsequent blind scoring by an AHA certified examiner.

#### Secondary Outcomes

The GMFM-66^27^ was used to evaluate the children’s gross motor function. The MA2^28,29^ was used to evaluate the unimanual performance of the more and less affected upper extremity in terms of movement range, accuracy, dexterity, and fluency. Each child’s MA2 and GMFM-66 test sessions were video recorded for subsequent blind scoring.

The Canadian Occupational Performance Measure (COPM)^30^ was used to establish and evaluate the children’s functional goals (defined by their parents) in terms of the child’s performance and parent’s satisfaction. The Pediatric Evaluation of Disability Inventory-Computer Adaptive Test (PEDI-CAT)^31^ was used to evaluate functional skills in the daily activities and mobility domains. All questionnaires were completed by the parents. For the secondary outcomes, *P* values should be considered as a reference for exploratory purposes.

### Procedures

#### Treatment Group: Early HABIT-ILE

Interventionists and physical or occupational therapists oversaw daily by experienced and trained HABIT-ILE supervisors provided the early HABIT-ILE intervention. Additionally, to ensure fidelity to the intervention, therapists participated in 1 to 2 days of training to familiarize themselves with the therapeutic concepts; they were subsequently guided by the supervision team throughout the study. Throughout all the study and sites, the same supervision team ensured the exclusive use of HABIT-ILE, as well as the adaptation of the intervention to the child’s age, motor abilities, and functional goals.

The young children participated in day-camp therapy 5 days a week over 2 weeks.^19,32,33^ At least 1 interventionist was assigned to each child. As described in the pilot study,^20^ to account for the specificity of children younger than 5 years, we modified the original HABIT-ILE protocol.^19^ Daily sessions consisted of 5 hours of HABIT-ILE per day with 3 hours in the morning, 2.5 hours off (nap/rest time), and 2 hours in the afternoon, for a total of 50 hours. Briefly, therapy activities are chosen according to the child’s baseline upper extremity and lower extremity capacities and postural control. The activities are progressed by varying the environmental constraints, moving to more challenging activities as performance improves.

#### Control Group: Spontaneous, Unstructured Motor Activity

Young children allocated to the active control group continued with their usual daily life activities, consisting mainly of spontaneous unstructured motor activity (at home or daycare). The estimated usual motor activity time of children 1 to 4 years old is around 5 hours per day.^34,35^ To ensure that this theoretical amount of hours reported for this age group in the literature matched the actual amount of activity performed by the children in our sample (time spent in movement),^36,37^ we measured the daily amount of activity using inertial sensors in both groups between T0 and T1. The children in the control group performed their usual therapies during this time, including physical, occupational, and psychomotor therapy (mean total, 2 hours per week).

#### Sample Size

As reported in the study protocol,^38^ we calculated the sample size based on previous studies of children older than 5 years^39^ and the pilot trial performed in children younger than 5 years.^20^ Those studies showed an improvement minima of 6 AHA units in the treatment group and of 2 AHA units in the control group (effect size, 1.26).^39^ The AHA improved by 10 (SD, 6.7) AHA units at the third month of follow-up.^20^ Consequently, a minimal improvement of 1 SD in the treatment group vs the control group was expected, with an α of .05 and a 1-β of 0.9. Accordingly, 46 participants were required (23 per group) but we planned to recruit 50 children in case of dropouts.

### Statistics

The analyses were performed by an independent group of statisticians using SAS/STAT software, version 9.4 (SAS Institute). As planned in the study protocol,^38^ between-group comparisons of the primary outcome were performed using analysis of covariance (ANCOVA) with adjustment for baseline measurements.^40^ Additionally, the same analyses were performed with consideration of age (older than/younger than 2 years old, ie, up to 35 and from 36 months) and manual ability limitations (Mini-MACS level) to determine their impact on the therapy outcomes. ANCOVA was also performed on the secondary outcomes. If homoscedasticity and normality were not met, nonparametric analyses (Wilcoxon test) were performed. For exploratory purposes, the paired *t* test (or Wilcoxon) was performed within groups to compare outcomes between assessment time points. The Fisher (or χ^2^) test was also used to comparing qualitative data. Effects were considered statistically significant at *P* < .05.

## Results

From a total of 140 young children screened, 90 were excluded: 13 had no CP diagnosis and 15 did not have UCP, 18 were older than 59 months, 16 had associated problems that could interfere with assessments/intervention, and 1 had a scheduled botulinum toxin injection during the study period. Twenty-seven children who fulfilled all the inclusion criteria could not be peer matched. Among the 50 young children included and randomized, 1 from the control group dropped-out at T0 due to COVID-19 restrictions. One child from each group did not perform the last assessment session (eTable 1 in Supplement 1).

The groups did not show imbalances at baseline in terms of age, lesion type, affected side, GMFM-66 score, or sex (Table 1). Only the Mini-MACS level showed a slight imbalance between groups. Also, there was no between-group imbalances in the number of minutes per week of usual rehabilitation, including physical therapy, occupational therapy, and psychomotor therapy (Table 1).

**Table 1.** Baseline Participant Characteristics

Characteristic	Control group (n = 24)	HABIT-ILE group (n = 25)
Sex, No. (%)		
Male	9 (38)	14 (56)
Female	15 (63)	11 (44)
Age, mo		
Mean (SD)	33.16 (12.16)	35.51 (11.99)
Median (Q1-Q3)	32.8 (25.4-44.3)	35.3 (24.7-45.5)
Range	14-52	16-54
Lesion type, No. (%)		
Premature	2 (8)	4 (16)
Perinatal asphyxia	1 (4)	1 (4)
Cerebrovascular accident	22 (92)	23 (92)
Other	2 (8)	3 (12)
Affected side, No. (%)		
Right	19 (79)	20 (80)
Left	5 (21)	5 (20)
GMFM-66, No. (%)		
I	16 (67)	17 (68)
II	5 (21)	5 (20)
III	2 (8)	1 (4)
IV	1 (4)	2 (8)
Mini-MACS, No. (%)		
I	1 (4)	7 (28)
II	16 (67)	16 (64)
III	5 (21)	1 (4)
IV	1 (4)	1 (4)
V	1 (4)	0
Physiotherapy, min per wk		
Mean (SD)	40.45 (11.84)	40.77 (9.25)
Median (IQR)	37.5 (30.0-45.0)	45.0 (30.0-45.0)
Range	30-60	30-60
Occupational therapy, min per wk		
Mean (SD)	44.58 (8.91)	45.00 (9.49)
Median (IQR)	45.0 (45.0-45.0)	45.0 (45.0-45.0)
Range	30-65	30-60
Psychomotor therapy, min per wk		
Mean (SD)	44.62 (13.76)	43.75 (8.66)
Median (IQR)	45.0 (40.0-60.0)	45.0 (42.5-45.0)
Range	15-60	30-60

Abbreviations: GMFM-66, Gross Motor Function Measure; HABIT-ILE, Hand-Arm Bimanual Intensive Therapy Including Lower Extremities; min, minutes; Mini-MACS, Manual Ability.

### Primary Outcome

**Figure 2.**
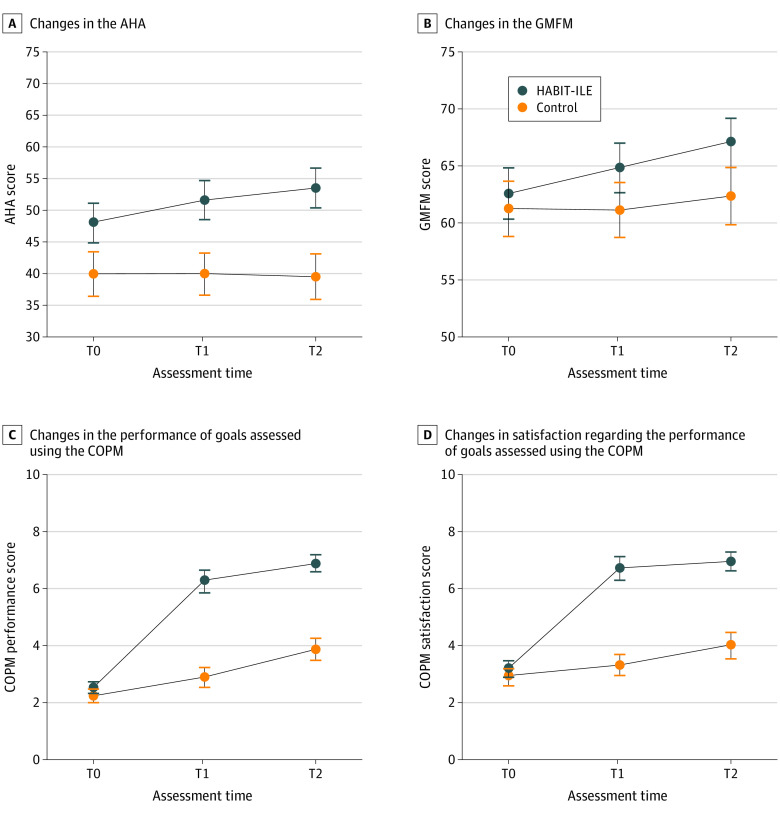
Changes in Motor and Functional Goals After Hand-Arm Bimanual Intensive Therapy Including Lower Extremities (HABIT-ILE) Training Blue dots indicate the mean of the treatment group (HABIT-ILE); orange dots indicate mean of the control group. For all dots, the whiskers represent the standard error. AHA indicates Assisting Hand Assessment; COPM, Canadian Occupational Performance Measure; GMFM, Gross Motor Function Measure; T0, first testing session (baseline); T1, second testing session 2 weeks after T0; T2, third testing session 3 months after T0.

Mini-MACS/AHA mean score differences (MD) between T0 and T2 were significantly larger in the HABIT-ILE than the control group (MD, 5.19; 95% CI, 2.84-7.55; *P* < .001) (Table 2). Larger differences were also found in the HABIT-ILE group between T0 and T1 but not between T1 and T2 (Figure 2 and Table 2). Subgroup analysis revealed greater improvements in children younger than 2 years old (Table 3) with no differences in the extent of the improvement between the different Mini-MACS levels (Table 3).

**Table 2.** Motor Assessments, Goals, and Questionnaire^a^

	Mean difference (95% CI)		
Outcome	Control group^b^	HABIT-ILE group^c^	ANCOVA, adjusted difference HABIT-ILE (control group)^c^
Primary outcome			
AHA ΔT2-T0, AHA units	0.39 (2.69)	5.17 (4.76)	5.19 (2.84-7.55)
AHA ΔT1-T0, AHA units	0.21 (3.40)	3.60 (4.30)	3.82 (1.55-6.10)
AHA ΔT2-T1, AHA units	0.09 (2.95)	1.58 (2.38)	1.45 (−0.20 to 3.10)
Secondary outcomes			
GMFM-66			
ΔT2-T0, logits, %	1.21 (3.84)	5.80 (3.26)	4.72 (2.66-6.78)
ΔT1-T0, logits, %	−0.09 (2.44)	2.84 (3.17)	3.01 (1.38-4.63)
ΔT2-T1, logits, %	1.18 (3.66)	2.97 (4.03)	1.75 (−0.58 to 4.07)
MA2 score of more-affected hand			
ROM			
ΔT2-T0, %	1.99 (6.50)	14.38 (13.29)	14.12 (8.26-19.97)
ΔT1-T0, %	−0.31 (16.53)	10.34 (15.70)	12.56 (3.56-21.56)
ΔT2-T1, %	2.47 (17.22)	3.77 (9.23)	0.75 (−7.55 to 9.05)
Accuracy			
ΔT2-T0, %	8.61 (11.50)	10.43 (13.25)	4.03 (−3.20 to 11.26)
ΔT1-T0, %	5.25 (13.70)	6.47 (15.38)	4.05 (−4.05 to 12.15)
ΔT1-T2, %	3.65 (17.22)	3.86 (10.84)	−0.75 (−9.51 to 8.02)
Dexterity			
ΔT2-T0, %	−0.74 (8.23)	15.85 (13.73)	18.18 (12.21-24.16)
ΔT1-T0, %	−0.80 (15.21)	10.21 (13.88)	12.61 (4.89-20.33)
ΔT2-T1, %	−0.36 (15.23)	5.98 (12.54)	6.16 (−2.20 to 14.51)
Fluency			
ΔT2-T0, %	5.83 (14.85)	12.71 (12.70)	8.77 (1.07-16.46)
ΔT1-T0, %	0.78 (14.47)	9.82 (14.60)	10.63 (2.66-18.60)
ΔT2-T1, %	4.19 (18.21)	2.88 (12.47)	−1.13 (−10.52 to 8.26)
MA2 score of less-affected hand			
ROM			
ΔT2-T0, %	−0.64 (8.41)	9.11 (11.56)	5.98 (1.45-10.50)
ΔT1-T0, %	−4.79 (17.14)	7.86 (16.97)	6.92 (−1.20 to 15.04)
ΔT2-T1, %	4.35 (18.37)	1.23 (11.69)	−1.03 (−10.18 to 8.12)
Accuracy			
ΔT2-T0, %	3.65 (10.78)	7.33 (17.64)	3.57 (−1.37 to 8.50)
ΔT1-T0, %	−2.00 (18.80)	4.96 (18.45)	6.83 (−1.25 to 14.91)
ΔT1-T2, %	5.74 (17.68)	2.17 (10.61)	−3.57 (−12.18 to 5.05)
Dexterity			
ΔT2-T0, %	0.17 (8.91)	9.49 (8.33)	8.93 (4.37-13.49)
ΔT1-T0, %	0.82 (8.39)	4.21 (9.42)	3.01 (−1.67 to 7.69)
ΔT2-T1, %	−0.69 (11.48)	5.23 (6.96)	5.92 (0.30-11.55)
Fluency			
ΔT2-T0, %	1.24 (17.18)	8.16 (11.61)	2.78 (−3.28 to 8.84)
ΔT1-T0, %	−2.98 (20.10)	6.88 (17.39)	5.36 (−3.24 to 13.97)
ΔT2-T1, %	4.35 (19.89)	0.99 (13.61)	−2.78 (−13.03 to 7.48)
COPM of children’s performance			
ΔT2-T0	1.22 (1.17)	4.82 (1.26)	3.62 (2.91-4.32)
ΔT1-T0	0.20 (0.57)	4.18 (1.67)	4.00 (3.27-4.73)
ΔT2-T1	1.03 (1.24)	0.65 (1.37)	−0.38 (−1.16 to 0.40)
ΔT2-T0	0.82 (1.47)	4.18 (1.84)	3.53 (2.70-4.36)
COPM parent’s satisfaction			
ΔT1-T0	0.07 (1.03)	3.86 (2.31)	3.97 (3.10-4.84)
ΔT2-T1	0.81 (1.19)	0.34 (1.69)	−0.49 (−1.36 to 0.39)
PEDI-CAT: daily activity			
ΔT2-T0, scaled score	0.83 (1.97)	1.96 (2.08)	1.40 (0.29-2.51)
ΔT1-T0, scaled score	−0.13 (1.60)	1.63 (2.22)	1.90 (0.79-3.02)
ΔT2-T1, scaled score	0.91 (1.98)	0.13 (2.17)	−0.52 (−1.73 to 0.70)
PEDI-CAT: mobility			
ΔT2-T0, scaled score	0.65 (1.58)	1.39 (2.59)	0.86 (−0.33 to 2.04)
ΔT1-T0, scaled score	−0.13 (1.73)	0.54 (1.86)	0.70 (−0.35 to 1.75)
ΔT2-T1, scaled score	0.70 (1.87)	0.88 (2.92)	0.30 (−1.16 to 1.76)

Abbreviations: AHA, Assisting Hand Assessment; GMFM-66, Gross Motor Function Measure; HABIT-ILE, Hand-Arm Bimanual Intensive Therapy Including Lower Extremities; MA2, Melbourne Assessment 2; ROM, range of motion; COPM, Canadian Occupational Performance Measure; PEDI-CAT, Pediatric Evaluation of Disability; T0, first testing session (baseline); T1, second testing session; T2, 3 months after T0.

^a^ Inventory-Computer Adaptive Test; Δ, score difference between assessment times.

^b^ *P* = .49.

^c^ *P* < .001.

**Table 3.** Changes in Assisting Hand Assessment as a Function of Age Group and Manual Ability Level^a^

Subgroup analyzed	Mean difference (SD)		ANCOVA, adjusted difference HABIT-ILE (control group)		Heterogeneity test, *P* value
	Control group	HABIT-ILE group	Mean difference (95% CI)	*P* value	
AHA ΔT2-T0, AHA unit					
Younger than 36 mo	−0.23 (2.80)	6.38 (5.91)	7.46 (4.33-10.59)	<.001	.04
Older than 36 mo	1.20 (2.44)	3.73 (2.45)	2.65 (−0.65 to 5.96)	.11	
Mini-MACS					
Level I, AHA ΔT2-T0, AHA unit	2.00 (0.00)	4.29 (2.29)	2.34 (−6.16 to 10.85)	.58	.25
Level II, AHA ΔT2-T0, AHA unit	0.07 (3.15)	4.80 (5.43)	4.74 (1.88-7.60)	.002	
Levels III-V, AHA ΔT2-T0, AHA unit	0.86 (1.57)	11.00 (1.41)	10.10 (3.70-16.49)	.003	

Abbreviations: AHA, Assisting Hand Assessment; ANCOVA, analysis of covariance; HABIT-ILE, Hand-Arm Bimanual Intensive Therapy Including Lower Extremities; Mini-MACS, Manual Ability Classification System for children with cerebral palsy aged 1 to 4; T0, first testing session (baseline); T2, 3 months after T0.

^a^ Classification system for children 1 to 4 years; level I includes children with minor manipulative limitations, if any; level V includes children with severe manipulative disabilities; Δ, score difference between assessment times.

### Secondary Outcomes

The results of the secondary outcomes have been presented for exploratory purposes. GMFM-66 MDs between T0 and T2 were larger for the HABIT-ILE than the control group (MD, 4.72; 95% CI, 2.66-6.78). This was also the case as between T0 and T1 but not between T1 and T2 (Figure 2 and Table 2).

MA2 MDs (more affected upper extremity) between T0 and T2 were larger for the HABIT-ILE than the control group for movement range (MD, 14.12; 95% CI, 8.26-19.97), dexterity (MD, 18.18; 95% CI, 12.21-24.16), and fluency (MD, 8.77; 95% CI, 1.07-16.46) but not accuracy (MD, 4.03; 95% CI, −3.20 to 11.26) (Table 2). The less affected upper extremity had MDs between T0 and T2 and were larger for the HABIT-ILE than the control group for movement range (MD, 5.98; 95% CI, 1.45-10.50) and dexterity (MD, 8.93; 95% CI, 4.37-13.49), but not for accuracy (MD, 3.57; 95% CI, −1.37 to 8.50) or fluency (MD, 2.78; 95% CI, −3.28 to 8.84).

COPM MDs between T0 and T2 were larger for the HABIT-ILE than the control group for children’s performance (MD, 3.62; 95% CI, 2.91-4.32) and parents’ satisfaction level (MD, 3.53; 95% CI, 2.70-4.36) (Figure 2 and Table 2). Scaled PEDI-CAT MDs were larger for the HABIT-ILE than the control group only for the daily activity domain between T0 and T2 (MD, 1.40; 95% CI, 0.29-2.51) (Table 2).

The activity count did not differ between the HABIT-ILE (n = 22) and the control group (n = 16) for the less affected upper extremity (HABIT-ILE: mean, 43.4 [SD, 8.43] activity count per second; control: mean, 39.7 [SD, 5.15] activity count per second; *P* = .28). In contrast, mean activity count was higher for the more-affected upper extremity in the HABIT-ILE (mean, 27.7 [SD, 5.3] activity count per second) than the control group (mean, 23.2 [SD, 2.61] activity count per second; *P* = .002) (eTable 2 in Supplement 1).

## Discussion

This multicenter RCT confirmed our hypothesis that 50 hours of early HABIT-ILE would improve bimanual performance more than usual unstructured motor activity in young children with UCP aged 1 and 4 years old. Moreover, improvements in the main outcome occurred in children who were younger than 2 years old. In addition, greater improvements in gross motor function, functional goals, and daily life activities occurred with HABIT-ILE than spontaneous unstructured motor activity.

The magnitude of change in bimanual performance measured by the AHA (5.17 AHA units) after 50 hours of therapy exceeded the smallest detectable difference (5.0 AHA units)^41^ and was similar to that observed in older children after 90 hours of HABIT-ILE (6 AHA units).^32^ Furthermore, the effect of the therapy was greater in the younger children than the older children included, although this needs to be confirmed by further studies. This large change in children younger than 2 years, despite the lower dose of HABIT-ILE than that provided to school-aged children (6 years old and older), suggests that this early intervention had a positive impact on neural structures, particularly the corticospinal tract, since intense structured activity has a large impact on the corticospinal tract during the early stages of development, as demonstrated in an animal model.^42^ The smaller effect in the older children in the present study suggests that more than 50 hours of intervention are required for children older than 2 years to achieve a clinically meaningful change. Such a change occurred in school-aged children who underwent 90 hours of HABIT-ILE. The secondary analyses of the AHA regarding the manual ability limitations through the Mini-MACS showed a larger improvement in children with greater limitations. These results are in line with reports indicating that children with greater limitations have lower AHA scores and their performance stabilizes at an older age.^43,44^ Therefore, the window of opportunity for progression of performance may be wider in this group.

Between-group differences were also found in gross motor function and unimanual performance. Previous studies in young children with UCP mainly focused on the more affected upper extremity.^45,46^ The results for unimanual performance and gross motor function in the present study suggest that the less affected hand and the lower extremities/trunk can be successfully trained concomitantly to the more affected upper extremity in these young children, with no loss of effectiveness on the more affected upper extremity. This is supported by a previous report^47^ of intensive interventions in school-aged children with UCP that found that the addition of the lower extremities/trunk component did not affect upper extremity performance. The larger activity count for the more affected upper extremity in the HABIT ILE than the control group highlights the importance of intensity, in terms of amount of active movement, on performance. However, although the activity count for the less affected upper extremity did not differ between groups, the unimanual performance of this upper extremity also improved more in the HABIT-ILE group, suggesting that the structured motor activities also contribute to the effectiveness of this intervention. This is in agreement with a previous longitudinal study showing the influence of therapy content on improvement in children with CP.^48^ In addition, most improvements in motor outcomes at the end of the HABIT-ILE were maintained at the 3-month follow-up (Tables 2, Figure 2), similarly to previous studies of this intervention in school-aged children.^32,49^ This finding suggests that participation in an intensive intervention at such a young age opens a window of opportunity to promote new motor improvements in the ensuing weeks and months, probably through the improvement in motor performance and motor learning abilities acquired during the therapy, as well as the motivation to learn new skills.

The changes in the PEDI-CAT and the COPM scores could indicate that HABIT-ILE positively impacted on the child’s daily life activities and the mastering of functional goals. The magnitude of the change in both outcomes was similar to previous studies in school-aged children with UCP after HABIT-ILE.^49^ In addition, the improvements found in the present study were greater than those found in studies involving only upper extremities training in young children with UCP.^46^ This highlights the importance of including gross motor function training in intensive interventions at this age to improve the performance of daily life activities, despite the sometimes lengthy time required to lead the parents and children through the goal definition process.

The functional changes observed in this study most probably result from neuroplastic changes induced by HABIT-ILE.^12,13^ These neuroplastic changes are probably relevant in children with CP because the persistent inflammation provoked by the early lesion^50^ alters a number of processes in the gray and white matter.^51,52,53^ Several features of the intervention likely potentiate neuroplastic changes, such as the motivating, child-friendly, enriched environment designed to promote motor experiences. In animal models, this type of stimulation improves cognitive, motor, and social function and is associated with morphological brain changes.^54,55,56^ In addition, HABIT-ILE promotes the acquisition of new motor skills.^12,13^ Several animal model studies have found changes in white matter, notably in the corticospinal tract, associated with the effects of motor training, in particular when the difficulty of the task is progressively increased during the training.^57,58^ Therefore, intensive therapies may promote activity-dependent neuroplasticity, avoid maladaptive changes, and stimulate adaptative neuroreorganization.

### Limitations

Despite our efforts to control for confounding factors by using the strictest method available, some factors, such as the cognitive level of participants, may have impacted our results. The activity count data of both upper extremities could not be analyzed in all the children of each group; however, the results seem to reflect the overall behavior of the study group. Another limitation could be the lack of evidence relating to other interventions using similar protocols with which to compare our results. Lastly, the results of our secondary outcomes should be considered as exploratory because we did not perform adjustment for multiple comparisons.

## Conclusions

This multicenter RCT provides new evidence supporting the effectiveness of HABIT-ILE provided as an early intervention for young children with UCP, as compared with spontaneous, unstructured motor activity. As recommended recently,^59^ the next step is to promote the integration of this type of early intervention into clinical guidelines. This would promote the use of therapies based on scientific evidence in the rehabilitation process of children with CP from a young age.

## Notes

Supplement 1. eTable 1. Baseline characteristics of participants lost at baseline and follow-up eDocument 2. Method of Motor Activity Count eTable 2. IMU measurement of the dominant and non-dominant limbs

Supplement 2. Study protocol

Supplement 3. Data sharing statement
