# Supplementary material for: Changes Induced by Early Hand-Arm Bimanual Intensive Therapy Including Lower Extremities in Young Children With Unilateral Cerebral Palsy: A Randomized Clinical Trial
Source: JAMA Pediatr. 2023 Nov 6;178(1):19–28. doi: 10.1001/jamapediatrics.2023.4809 (PMC10628844; doi:10.1001/jamapediatrics.2023.4809)
Supplement: Supplement 1. — eTable 1. Baseline characteristics of participants lost at baseline and follow-up eDocument 2. Method of Motor Activity Count eTable 2. IMU measurement of the dominant and non-dominant limbs [file jamapediatr-e234809-s001.pdf]

# Supplemental Online Content

Araneda R, Ebner-Karestinos D, Paradis J, et al. Changes induced by early Hand-Arm Bimanual Intensive Therapy Including Lower Extremities in young children with unilateral cerebral palsy: a randomized clinical trial. *JAMA Pediatr*. Published online November 6, 2023. doi:10.1001/jamapediatrics.2023.4809

**eTable 1.** Baseline characteristics of participants lost at baseline and follow-up

**eAppendix.** Method of Motor Activity Count

**eTable 2.** IMU measurement of the dominant and non-dominant limbs

This supplemental material has been provided by the authors to give readers additional information about their work.

.

© 2023 Araneda et al. *JAMA Pediatr.*

**eTable 1. Baseline characteristics of participants lost at baseline and follow-up**

| Characteristics | | Control group (n=2) | | HABIT-ILE group (n=1) |
|---|---|---|---|---|
| | | Lost at T0 | Lost at T2 | Lost at T2 |
| Gender | Male / Female | Male | Female | Female |
| Age | Months | 47 | 48 | 40 |
| Lesion Type | Cerebrovascular accident, yes/no | Yes | Yes | Yes |
| Affected Side | Right / Left | Right | Left | Right |
| GMFCS-ER | Level I | | Yes | Yes |
| | Level II | Yes | | |
| Mini-MACS | Level I | | | |
| | Level II | Yes | Yes | Yes |

Abbreviations: HABIT-ILE, Hand-Arm Bimanual Intensive Therapy Including Lower Extremities; GMFCS-ER, Gross Motor Function Classification System-Expanded and Revised; Mini-MACS, Manual Ability Classification System for children 1-4 years

## eAppendix. Method of Motor Activity Count

### Participants

To compare activity levels between groups, as well as between the less and more affected upper extremities (UE), 50 children were included in this analysis.

### Material

The Physilog 5® is a Swiss-made, wearable and versatile inertial sensor (3D accelerometers, 3D gyroscopes).

### Procedure

The children wore a Physilog 5® on each wrist between 9 am and 5 pm for a minimum of 5 days between T0 and T1 (during the HABIT-ILE camp for the treatment group and during daily life for the control group).

For quality check purposes, a paper-based log of activities was completed during the same period (T0-T1) for children in the treatment and control groups, by both therapists and parents.

### Data analysis

Activity count computation was based on a simple estimation of the amount of acceleration per time unit. 3D IMU 128Hz data were first filtered with a sixth-order bandpass filter to remove the gravity constant component. Then, for each 1s batch, the mean of each acceleration axis was divided by the activity count ratio (r =0.001664 g/AC) to obtain an activity count estimation per axis. The norm of these 3 axis values corresponds to the activity count, for the given 1s batch[1]. The AC (less affected UE) and AC (more affected UE) are respectively the activity count of the less affected UE and the activity count of the more affected UE.

© 2023 Araneda et al. *JAMA Pediatr.*

Data of children were included in the analysis if they had 4 days of recording for the 2 sensors. Days during which the children were inactive for more than 50 minutes were removed from the analysis. To avoid bias due to different nap times in the control group, the analyses were conducted on 3 hours in the morning, except for the first 15 minutes to allow time to adapt to wearing the sensors.

### Statistical analysis

After testing the normality of the data distribution with the Shapiro wilk test, the 2 groups were compared using parametric tests.

For each child, outliers (value 1.5 times greater or less than the IQR) were removed. The mean activity count was computed on the remaining days. After testing the normality of the data distribution with the Shapiro wilk test, AC (less affected UE) and AC (more affected UE) were compared between the 2 groups using appropriate tests.

### Inertial sensor metrics

The mean activity of the less affected UE was greater in the treatment group, but this difference was not significant. The mean activity of the more affected UE was greater in the treatment group and this difference was significant (eTable 2).

## eTable 2: IMU measurement of the dominant and non-dominant limbs

| MEASURE | TREATMENT GROUP (n=22) | CONTROL GROUP (n=17) | P-value |
|---|---|---|---|
| AC (less affected UE) ($AC.s^{-1}$) | 43.4 (8.4) | 39.7 (5.2) | 0.284[d] |
| AC(more affected UE) ($AC.s^{-1}$) | 27.7 (5.3) | 23.2 (2.6) | 0.002[e] |

The AC (less affected UE) and AC (more affected UE) are expressed as mean (standard deviation) [d]Mann-Whitney U test, [e]Welch’s test.

© 2023 Araneda et al. *JAMA Pediatr.*
