# Supplementary material for: Changes Induced by Early Hand-Arm Bimanual Intensive Therapy Including Lower Extremities in Young Children With Unilateral Cerebral Palsy: A Randomized Clinical Trial
Source: JAMA Pediatr. 2023 Nov 6;178(1):19–28. doi: 10.1001/jamapediatrics.2023.4809 (PMC10628844; doi:10.1001/jamapediatrics.2023.4809)
Supplement: Supplement 2. — Study protocol [file jamapediatr-e234809-s002.pdf]

# Proposal application form Proposal Template

## A. Basic project data

- Project Title:

**Functional, neuroplastic and biomechanical changes induced by early Hand and Arm Bimanual Intensive Therapy Including Lower Extremities (HABIT-ILE) in pre-school children with uni-and-bilateral cerebral palsy: a European project**

- Project acronym: **Early HABIT-ILE**

- Duration of the project (months): 48 months

- Project Summary (max. 4000 characters including spaces)

An urgent priority in the field of paediatric neurorehabilitation is the development of **effective early motor interventions**. Animal studies strongly suggest early intensive rehabilitation may have a large impact on brain and corticospinal organisation, reducing motor impairments and their mid- to long-term consequences in children with cerebral (CP). Although most brain growth, development and organization occurs within the first 2 years of life, the majority of research into the effectiveness of intensive rehabilitation and mechanisms that underlie responses to therapy has been conducted in school-age children (i.e. > 6 years). Furthermore the main focus of research has been on children with unilateral CP, despite the fact that bilateral CP is the most prevalent form. **Studies of the effectiveness of early intensive interventions on global motor function in larger groups of children with CP, including bilateral CP are thus required.**

Hand and Arm Bimanual Intensive Therapy Including Lower Extremities (**HABIT-ILE**)[1] applies the concepts of motor skill learning and intensive training to both the upper (UE) and lower extremities (LE) and has been shown to improve aspects of motor function of the UE and LE in school age children with unilateral[2] and bilateral[3] cerebral palsy across the 3 levels of the International Classification of Functioning, Disability and Health (ICF). **The principles and content of HABIT-ILE can be applied to pre-school children and this method is highly promising for early rehabilitation.**

The primary objectives of this study are to evaluate the effect of **two weeks of early HABIT-ILE on bimanual performance in** pre-school children with unilateral CP (Randomized Controlled Trial 1=RCT1, N=50 children) and on **gross motor function** of children with bilateral CP (RCT 2, N= 50 children), in comparison with two weeks of **usual motor activity including usual rehabilitation (control group)**. The primary outcome measures will be the difference in Assistive Hand Assessment (**AHA**) and Gross Motor Function Measure (**GMFM**) between base line and at three months.

The network involved in the Early HABIT-ILE project will provide a means for rapid dissemination of the results to clinical centres in French speaking countries, Europe and then world-wide. The early increase in autonomy and reduction of the mid-term consequences of CP is very likely to reduce **the economic cost and societal impact of "cerebral palsy"** at the level of the health care systems. Furthermore, the model of early intensive therapy proposed differs greatly from current rehabilitation standards in many countries. The results of this study should provide high evidence for changing, not only current models of motor rehabilitation in children with CP, but **health policies**, leading to a **new era of early rehabilitation for children with CP.**

### 1. Proposal's context

Cerebral palsy (CP) affects between 2 and 3.6 out of 1000 live births[4]. It causes abnormal patterns of movement and posture, and cognitive and sensory function may also be impaired[5,6]. These impairments result from structural abnormalities in the brain that occur at different periods of brain development, affecting different processes such as neuronal migration, differentiation, neuronal growth and myelination[7,8]. Therefore, the timing, extent and location of the central damage[9,10] impact the subsequent global functioning

of the brain. Symptoms are generally apparent before 18 months of age and the diagnosis is generally confirmed between 13 and 19 months of age[11]. Because CP-related motor deficits have an impact on activity level and autonomy[11,12], particular attention has been paid over the last 15 years to interventions for the treatment of motor dysfunctions. **The development of effective early motor rehabilitation is considered an urgent priority in the field of pediatric neurorehabilitation.** Intensive, activity-based, goal-directed interventions have been shown to effectively improve motor function in school-age children with CP[13]. **Although the major brain growth, development and organization occurs within the first 2 years of life, the majority of research into the effectiveness of intensive rehabilitation and mechanisms that underlie responses to therapy has been conducted in school aged children (i.e. > 6 years**)[11]. It is thus crucial to evaluate the effects of intensive rehabilitation in pre-school children, both at a functional and at a cortical level since animal models suggest that such interventions may prevent the development of secondary damage, positively impacting the whole developmental curve of the children.

Animal models of cerebral palsy have shown that the impairments that occur following perinatal brain injury are mainly the result of persistent inflammation[14-16], causing alterations in neurogenesis, axonal growth and synaptogenesis, as well as an important deficit in the myelination of white matter due to an alteration in the oligodendrocyte and its precursors[16,17]. These white matter impairments are likely the cause of a wide range of brain dysfunctions in children with cerebral palsy and contribute to alterations in neuronal activity[18,19]. **Animal studies have suggested that the generation and maturation of oligodendrocytes is activity dependent** and that the acquisition of a new motor skill increases the generation of oligodendrocytes and oligodendrocyte precursors only 2 hours after the initiation of the motor training[20,21]. This remarkably fast oligodendrocyte production, along with the rapid increase in the number and size of dendritic spines (synaptic plasticity) with activity[22,23], **suggest that an intervention based on motor skill learning may contribute to improve white matter myelination and thus brain function.**

The organization/**structure** of the corticospinal tract (CST) is also affected by the initial brain lesions, with persistence of ipsilateral projections rather than the development of contralateral projections[24-28], likely due to the lack of motor activity of the paretic limb. Rodent model studies have shown that motor skill learning modifies the white matter fractional anisotropy[20,29], **meaning that the improvement in the quality of the CST is also activity-dependent**[30,31]. Previous studies based on the timing of motor evoked potential response in babies suggest that the CST system is organized during the first two years of life[32]. **This time period may thus be a window of opportunity during which early stimulation may positively impact CST reorganization**[32]. These topographic changes are due to activity-dependent competition between developing cortico-spinal terminals and other spinal neural systems[33]. This damage secondary to the initial brain lesions can be reversed in kittens by providing intensive motor skill training to the impaired forelimb. **If provided in a window of opportunity, intensive use of affected forelimb in kittens drives a rebalance in the development of synaptic projections**[34]**.** In human beings the window of opportunity during which these projections might be rebalanced through motor stimulation is not well defined. It is generally assumed to occur during early infancy, probably until the age of two[33], during the main period of CST fibre myelination[35]. **These arguments from animal and human studies suggest that early motor skill learning based interventions should allow better development of CS projections and thus prevent secondary motor damage at the level of the CST.**

**Compared to the available literature in animals, only a few motor intensive intervention trials have been carried out in very young children and, to our knowledge all have focused on children with unilateral CP**. They evaluated modified forms of constraint-induced movement therapy (CIMT). An adapted baby-CIMT procedure for infants (<12 months) was proposed by Eliasson and colleagues[36]. Another adapted CIMT trial was performed in children between 2 and 3 years of age[37,38]. In addition, 2 research teams tested the effectiveness of CIMT in children with unilateral CP between the ages of 1 and 6 years[39,40] and a research team tested the feasibility of carrying out bimanual training in children aged 2 to 4 years[41]. The results of these studies provided moderate evidence about the application of CIMT or bimanual training in young children with unilateral CP. Higher daily intensities of training (Taub et al.[39] and DeLuca et al.[40]: 6 hours per day for a total of 60 hours) seemed to induce larger changes than lower intensity training (Eliasson et al.[36] and Nordstarand et al.[38]: 30 min per day, 6 days a week, for a total of 36 hours; Ferre et al.[41]: 2 hours a day, 5 days a week for 9 weeks), though comparison of results is hindered by differences in the ages of the samples and the use of different assessment tools. All the interventions proposed focused solely on the upper extremity (UE) of children with unilateral CP[39,42], despite the fact that the lower extremity (LE) is commonly affected. **The next step in the development of early intensive interventions is to evaluate the effectiveness of therapy which targets global motor function, including the lower extremities, in larger group of children with CP, including bilateral CP.**

Yannick Bleyenheuft (member of the project consortium) and her team designed an intervention for school-age children with unilateral and bilateral CP a few years ago to address both UE and LE deficits. **Hand and Arm Bimanual Intensive Therapy Including Lower Extremities (HABIT-ILE)[1] applies the concepts of motor skill learning and intensive training to both the UE and LE.** It involves stimulation of the UE and LE during combined activities for many hours each day for a period of 2 weeks. **This method has been shown to improve aspects of motor function of the UE and LE in children with unilateral[2] and bilateral[3] cerebral palsy across the 3 levels of the International Classification of Functioning, Disability and Health (ICF)**: body function and structures (e.g. strength and dexterity), activity (activities of daily living) and social participation (achievement of personal goals). In addition, recent findings indicate that intensive motor interventions such as HABIT-ILE have a positive impact on executive functions of school-age children with CP (Araneda et al., work in process). Furthermore, HABIT-ILE has been shown to improve the quality of CST fibers in children with CP (increase in the fractional anisotropy values) (Bleyenheuft et al., submitted)[43]. Moreover, the increase in fractional anisotropy was correlated with motor improvements in the hand[43]. **The principles and content of HABIT-ILE can be easily adapted and applied to pre-school children and this method is highly promising for early rehabilitation.**

**To summarize, animal studies strongly suggest early intensive rehabilitation may have an important impact on brain and corticospinal organisation, reducing impairments in children with CP. Studies that target both the UE and LE in pre-school children with both unilateral and bilateral CP are lacking and the timing of the window of opportunity for interventions to prevent secondary damage in children is yet unknown. A more accurate understanding would lead to guidelines for early rehabilitation.**

*Summary table of persons involved in the project:*

| Partner (Short name) | Name | First name | Current position | Involvement (person.month)* | Role & responsibilities in the project (4 lines max) |
|---|---|---|---|---|---|
| 1 (SB) | Brochard | Sylvain | Professor (Full), University Hospital Of Brest (France), Head of the pediatric rehabilitation department | 14.4 | -Project leader<br>-promotion/ communication and publication coordination<br>-Standardization, analysis and publication of the Biomechanical measures (WP3 leader)<br>-Inclusion of 12 children in Brest |
| 2 (YB) | Bleyenheuft | Yannick | Professor (Full), University of Louvain (Belgium) | 12 | -Standardization and implementation of the early HABIT-ILE across the 4 locations<br>-Responsible for functional, activities and participation outcomes<br>-Inclusion of 24 children<br>-Publication of the main and secondary functional outcomes & DTI results |
| 3 (AG) | Guzzetta | Andrea | Professor (Associate) | 7.2 | -Inclusion of 24 children -Standardization of the brain imaging data across the 4 locations<br>-Coordination of the brain imaging analysis according to expertise<br>-Publication of the connectivity outcome |
| 4 (MD) | Dinomais | Mickael | Professor (Full), University Hospital Of Angers (France), Head of the pediatric rehabilitation department | 4.8 | -Inclusion of 12 children in Angers<br><br>-Standardization of the brain imaging data across the 4 locations<br><br>-Analysis and publication of the morphological analysis |
| 5 (SS) | Sizonenko | Stéphane | Professor (associate),Geneva University Hospital (Switzerland) | 7.2 | -WP4 leader<br>-Experiments, analysis and publication of the results of the RCT on rodents (window of opportunity) |
| 6 (CN) | Newman | Christopher | PD, University Hospital of Lausanne | 4.8 | - Standardization and analysis of Activity measures of Children using sensors<br>- Standardization and analysis of Activity measures of rodents using sensors<br>- Publication of the activity outcome |

| | | | | | |
|---|---|---|---|---|---|
| 7 (IR) | Riquelme | Inmaculada | Professor of the department of Nursing and Physiotherapy at the University of the Balearic Islands | 0.5 | Standardization, analysis and publication of the pain outcomes |
| 8 (GL) | Legall | Grégoire | Professor (Full), University Hospital Of Brest | 0.5 | -Methodology throughout the project<br>-Coordination of the statistical analysis |
| 9 (EN) | Nowak | Emmanuel | Head of the data analysis center of CHRU Brest | 0.5 | -Statistical analysis coordination |

## 2.3. Research Plan

*Participants*

**One hundred** pre-school children with cerebral palsy (**50 unilateral** for RCT1 and **50 bilateral** for RCT2) will be included. Based on previous intensive motor skill learning trials in school-age children, the **inclusion criteria** will be: children with cerebral palsy (spastic or dyskinetic), unilateral or bilateral cerebral palsy, age 1 to 4 years inclusive (corrected age if preterm birth) and ability to follow instructions and complete testing according to the age. **Exclusion criteria** will be epilepsy, botulinum toxin injections or orthopedic surgery within the past six months or during the study period, as well as severe visual and cognitive impairments that could interfere with treatment/testing, usual contraindications to MRI (Metal implants, etc.), and parent(s) unable/inapt to provide consent for their child's participation. Based on their current list of children followed in each centres, there will not be any issue to recruit these group children in the 4 investigating centres of the project.

*Design and Randomization*

This project will involve **2 single-blind randomized controlled trials (RCTs), one with children with unilateral CP and the other with children with bilateral CP**. The decision to carry out two separate RCTs was made by the consortium and methodologist based on the fact that the causes, and most importantly for this project, the functional motor outcomes and rehabilitation goals differ for these two CP types. For each RCT, the randomisation will be carried out 24 hours per day using centralised computer randomisation by internet. A **pair-matched randomization** will be used in order to ensure comparability. For children with unilateral CP, each child will be matched with a child with the closest profile based on age (4 categories: 1,2,3,4 yo), the affected side (right or left), and the origin of the CP (prematurity, perinatal asphyxia, stroke, others). For children with bilateral CP, each child will be matched with a child with the closest profile based on age (4 categories: 1,2,3,4 yo), the Gross Motor Function Classification System level [55] (GMFCS, 3 categories: I-II, III-IV and V) and the origin of the CP (prematurity, perinatal asphyxia, stroke, others). All the efforts will be made in both RCTs to find the closest profile within the same year, same GMFCS level and same origin of CP. Group allocation will be then randomly determined within each pair. This design was chosen to limit the heterogeneity in clinical pattern between the intervention and control arms, while keeping the benefit of individual randomization. It is commonly used and widely accepted in the field of neuropediatric rehabilitation[56]. The 2 trials will also be equilibrated according to the age groups 1-2 (50% of the group) and 3-4 (50% of the group) in order to investigate the effect of window of opportunity.

In both RCTs (unilateral and bilateral), the children will be randomized to receive HABIT-ILE or the Control condition. The children in the control group will receive the therapy after the 3 month follow-up assessment in order to decrease drop-out at follow-up in this group, however, this will not be included in the analysis. Thus the between group comparisons will be carried out between T0 and T+15 and between T0 and T+90. The design of RCTs 1 and 2 is presented in Annex.

The HABIT-ILE group (unilateral n=25/bilateral n=25) will receive 50 hours of treatment over 2 weeks (5 hours per day of treatment, 5 days a week) whereas the control group (unilateral n=25 /bilateral n=25) will follow their usual activities for 2 weeks (nursery, usual therapy etc.).

*HABIT-ILE procedure*

HABIT-ILE[1-3] is a **motor skill learning-based therapy carried out in a camp-setting, with structured tasks of increasing motor difficulty and functional activities that require the use of both hands whilst sustaining postural or locomotor activity of the LE**. Tasks are based on games and the whole environment is set up such that the children perceive the camp as fun. For both the children with unilateral

and bilateral CP, the tasks will be chosen according to individualized functional goals previously defined by the parents (e.g drinking by him/herself, walking without help, opening a box of toys, holding a book, putting off pants, playing in sitting without support, etc.). Results of the initial assessments (T0) will be used to determine appropriate tasks for each child. Over the course of the 2 weeks, tasks will be progressed to further challenge bimanual coordination, postural control and LE function. Initially, the more affected upper extremity (UE) will be used as a passive stabilizer, with progressive encouragement towards more complex (active) use of this hand by introducing games that require more skilled use of the more affected UE. LE/postural involvement in the different tasks/activities will also be progressively more demanding: 1) sitting activities (initially on a chair or a mat potentially with a back support, progressing towards unsupported sitting and sitting on unstable supports e.g. roller or ball), 2) transitions from lower to higher postures using the UE for support, 3) stable gross motor activities (from standing with UE support to playing during free standing) and 4) movement activities (crawling, walking, running or jumping), with graded difficulty. More skilled/challenging UE activities will be systematically introduced in stable sitting and the child will be subsequently challenged to perform the UE activity in more challenging LE conditions. More challenging LE/postural conditions will also be progressively introduced while performing a bimanual task that has already been mastered.
Although the basic principles of HABIT-ILE will be followed[1], the toys, tasks and activities will be adapted so that only bimanual use will be possible. Babies and very young children can perform bimanual activities (bimanual reaching, holding, transfer, symmetrical and asymmetrical actions) at one year old[57]. However this requires the use of specific toys that afford bimanual use through their characteristics: e.g. for bimanual reaching / holding, use of a tool that is too big, too heavy or not stable enough to be picked up with one hand. The duration of activities as well as LE/postural associations will also be adapted to each child depending on his/her functional objectives, attentional ability and gross motor development. The whole process will be performed in a **child-friendly, fun environment adapted to the age of the child**.
**Eight children will undergo rehabilitation together**. Some activities will be carried out in groups and others individually. Each child will have his/her own therapist (PT or OT) as well as a student therapist working with him/her. The aim is to maintain a structured motor engagement time of 80 % throughout the 10 days of therapy. The post-doctoral scientist dedicated to the project will supervise all camps in order to standardize the procedures and train all therapists and students.
Since previous intensive treatments delivered by parents or at home with videos/online supervision have so far been disappointing, both regarding compliance with guidelines and the moderate motor changes observed[58], the highest feasible intensity treatment will be delivered in this study. **Recent studies on dosage support that 50 to 60 hours of intensive therapy are needed to induce motor changes[59,60]**. Taking into account usual motor activity time[61] and rest time (e.g. naps), 5 hours per day for a total of 50 hours is the best compromise between current scientific evidence on dosage and the number of feasible hours in this age group. This amount of therapy time is close to the CIMT trial that has provided the greatest effectiveness[39].
**The adaptation of HABIT-ILE to pre-school children is based on the know-how developed in school-age children** and a pilot camp will be performed in the second trimester of 2018 in Brussels in pre-school children (prior to the start of this project). The aim of the pilot camp is to develop and test a standardized therapeutic procedure before implementation in the multicentre study in Brussels, Pisa, Brest and Angers.

*Control procedure*

The control procedure consists of the child's usual activity, including nursery, usual rehabilitation, etc. The spontaneous activity of preschool children is usually oriented towards the discovery of their environment. A recent study based on accelerometers[61] demonstrated that toddlers spend around 7.6 hours/day carrying out motor activities. Since a proportion of these activities are performed at home, before or after going to the nursery/preschool (i.e. dressing in the morning, taking a bath, having dinner or brushing teeth before sleeping), excluding nap time, thus around 5 hours of daily motor activity were estimated to be carried out in nursery. The **content of the activity** will be documented by the person in charge (notebook) and the **amount of activity will be documented objectively by wearable sensors** (see WP 3).This control 'intervention' condition was chosen as the comparison group because it represents the current model of rehabilitation in many countries. The choice of **this control group is highly clinically relevant because it compares a new model of rehabilitation to the current on-going model of rehabilitation in many countries**. It also allows the comparison of the "structuration" of motor activities and "spontaneous activities" to be carried out.

*Evaluation criteria*

In each RCT, both groups (N=25) will be assessed 3 times: T0, T+15 days and T0+90 days (See study design in Figure 4).

For the RCT 1, the primary outcome measure will be the difference in the **Assisting Hand Assessment[62] (AHA, from 18 months to 18 years) or Mini Assisting Hand Assessment[63]** (Mini-AHA, from 8 to 18 months) between T0 and T0+90 days. It quantifies through a Rash analysis the assistance that the more affected hand can provide to the less affected hand in bimanual activities. This primary outcome was chosen because 1) it has become the gold standard measure of bimanual performance in children with unilateral CP 2) its **responsiveness, reliability and validity** have been demonstrated in children with CP including infants and toddlers[62]. 3) its raw scores are transformed through a rash-model into linear measures, allowing the use of parametric statistics 4) the assessment can be **videotaped and scored by a trained examiner totally blind** to group assignment and timing of assessment ensuring a blind scoring, which is planned for this study. The minimal detectable change is 4 AHA-Units (0.97 logits) [62,64,65].

For the RCT2, **the primary outcome measure will be the difference in Gross Motor Function Measure GMFM (in% of logits) between T0 and T0+90 days**.The GMFM is a clinically validated tool designed to evaluate changes in gross motor function over time or following an intervention in children with cerebral palsy. It includes 66 items identified through **Rasch analysis** that best describe gross motor function in children with cerebral palsy of varying abilities. The items cover a large spectrum ranging from activities in lying and rolling to walking, running and jumping. This primary outcome was chosen 1) because it can be performed both in preschool and school-age children, allowing a sample size calculation based on previous data in school-age children; 2) its **responsiveness[66], reliability and validity** have been demonstrated in children with CP[67,68] including infants and toddlers[69]; 3) its raw scores are transformed through a rash-model into linear measures, allowing the use of parametric statistics[70]; 4) the assessment can be **videotaped and scored by a trained examiner totally blind** to group assignment and timing of assessment ensuring a blind scoring, which is planned for this study. The blinded examiners of the consortium will be trained and their inter-examiner reliability will be tested by scoring videos of the same children. Finally, the developmental curves for the GMFM-66 are known for children with different levels of GMFCS allowing **significant clinical change to be differentiated from the normal physiological evolution of the child[71].** The minimal detectable change is 2 logits[66].

**The secondary outcomes will be investigated at T0, T+15 days and T0+90 days and are specified in the description of each work-package.** The **3 levels of the International Classification of Functioning, Disability and Health (ICF)** will be systematically evaluated for each child in order to track changes at the body level (e.g., assessing manual ability), activity level (using questionnaires) and at the level of social participation (using questionnaires)(See WP 1,2,3). **Although the number of assessments might seem quite high for children of this age, the estimated time to carry out the full assessment is< 2 hours for the functional assessment of the GMFM/AHA + evaluation criteria of WP1, 30-45 minutes for WP2 (MRI) and 30-45 minutes for WP3 (3D Movement analysis). The evaluation will be carried out over a day to allow the children to have free activities in between each test.**

*Statistical analysis: sample size and statistical analysis of the main primary criteria*

Unilateral CP (RCT1): The sample size was calculated based in one study performed in infants and young children (Eliasson & al., 2011)[37]. This study evaluated the effect of eco-Constraint Induced Movement Therapy (Eco-CIMT) using a cross-over design. They showed an improvement of 6 units in the AHA (Main outcome, Minimal detectable change of 4 AHA-unit) for the intervention group and a change of 2 units in the control group. In our study, we expect at least an improvement of 6 units after HABIT-ILE in a similar population in term of age. In addition, the effect size estimated in Eliasson's study was of 1.26 unit. With these results, and with an a=0.05 and a 1-ß=0.9, a sample size of 15 participants per group would be required, with a total of 30. The results obtained by Eliasson[37] are consistent with the results of our pilot study evaluating HABIT-ILE in 10 children with unilateral CP (ages between 1 and 4 years old), where the mean improvements observed in the AHA at 3 months was of 10 units with a SD of 6.7 (Personal data). To take into account the differences between the populations and interventions and to be cautious regarding the variability of our targeted population, our hypothesis is an effect size of at least 1 unit (i.e., an improvement of 1 SD in the HABIT-ILE group when compared with the control group) with an α =0.05 and a 1-ß=0.9. Therefore, for the sample size, 23 participants per group are required, with a total of 46. Considering potential drop-outs, **50 participants will be included in total.**

Bilateral CP (RCT2): The sample size was calculated based on the mean improvement observed in the GMFM in Bleyenheuft et al., (2017)[3], where the intervention group (HABIT-ILE) showed an improvement of 7 logits (Minimal detectable change=2 logit) and the control group showed a change of 2 logits in the same time period (3 months). To take into account the differences between the populations and to be cautious regarding the variability of our targeted population (younger pre-school infants), we consider that the SD of

improvement could reach at the most 5 logits. Then, we hypothesize a difference between both groups of 5 logits and a SD of improvement of 5 logits with an α=0.05 and a 1-β=0.9. With these hypothesis, a sample size of 23 participants per group is required, with a total of 46 participants. Considering potential drop-outs, **50 participants will be included in total**.

Improvements in GMFM and AHA will be compared between study groups using analysis of covariance (ANCOVA) to adjust for baseline measurement (as described and recommended in Vickers et al.[72]) Non-parametric methods will be performed whenever ANCOVA assumptions (homoscedasticity, normality) are not met. Age-subgroup (or according to other characteristics) and interaction analyses will be performed for exploratory purpose. As recommended by Good Clinical Practice, exploratory analyses will be used for hypothesis generating but cannot be conclusive alone. The statistical analysis of the secondary outcome measures are described in the relevant WPs. Secondary outcome analysis, as well as subgroup analysis, should be considered as exploratory unless using specific methods in order to control the Type I error probability (multiplicity problem).

*Quality procedure*
A clinical research assistant employed by the promoter will ensure the quality of the study, the data collection, documentation and recording etc., in accordance with the Standard Operating Procedures in Brest CHRU, Good Clinical Practice and current legislation. A web-based interface will be created for randomization, and an electronic case report form will be set up to collect all study data.

## 3. Impact and benefits of the project

*At the level of children with CP involved in the project and their families*

HABIT-ILE is a motor skill learning based intervention whose effect has been demonstrated in school-age children with both uni and bilateral CP. The main strength of this method is that it targets both the activity and participation levels of the ICF thus producing real-life, useful changes for the children, that **parents can perceive**. Thus **for the 100 participating European preschool age children who will undergo the intervention, we expect large functional, neuroplastic and biomechanical changes that will impact their whole developmental curve, improving functional ability, autonomy and participation in the short-, mid- and long-term.** Based on current evidence in children and animal models, we believe that the change in motor capacity, and thus the prevention of the mid-and-long term consequences of CP, will be greater than if than if the therapy was provided later, for instance at school age as it is currently the case in some countries.

*At the scientific level*

Validation of the effectiveness of early intensive motor therapy and definition of the best window of opportunity to promote the largest changes is a crucial step for the development of optimal rehabilitation in CP. A negative result would avoid false beliefs, avoiding unnecessary intensive treatment. A positive result for RCTs 1 and/or 2 would lead to a **turning point in the rehabilitation of individuals with CP as well as in the wider field of pediatric neuro-rehabilitation**. The main strength of the design of the early HABIT-ILE project (Inclusion criteria, paired randomisation, main outcome measure) is that the results will be relevant for children with **UNI and BILATERAL CP of all causes (Prematurity, Stroke, Malformations,…):** the results will thus impact **the whole community of individuals with CP** as well as the whole community of scientific and care professionals involved in CP**.**

*Dissemination*

Although training is required before HABIT-ILE can be implemented, it is a standardisable therapy that can be replicated in all clinical centres. In fact, HABIT-ILE has already become a reference method for school age children in many different countries such as Australia (CP Alliance) and Spain (Madrid). It has also **been implemented in France for the first time in a clinical setting** (Brest, July 2017). In order to disseminate HABIT-ILE, and intensive therapy in general, Yannick Bleyenheuft and her team developed a **specific training** (university certificate in Université Catholique of Louvain) program in 2013. So far 50 therapists (mostly physiotherapists and occupational therapists) have been trained and we plan to train 8 more therapists during the 4 years of the project. Moreover, the entire PT and OT departments of each participating centre will be involved in the project, aiding the promotion of evidence-based interventions. Since we hope to observe large changes in motor function, and to develop new clinical recommendations/guidelines for the treatment of children with CP, we believe that involving therapists from the beginning will encourage the transfer of practice from the study to routine care. **Thus the results of the Early HABIT-ILE project will be**

**rapidly disseminated through this network to clinical centres** in French speaking countries, Europe and then world-wide. In order to reach more people **clinical guidelines for the implementation of early HABIT-ILE will be written in English, French and other languages** according to the needs and possibilities.

*At the health care system level*

If the results are positive and the dissemination as large as we expect, then an impact can also be expected at the level of the health care systems of the different countries that implement the therapy in a clinical setting. The early increase in autonomy of each child and reduction of the mid-term consequences are very likely to **reduce the economic cost of “cerebral palsy”** for health systems.

Furthermore, the model of early intensive therapy proposed differs greatly from rehabilitation in many European countries. For instance, in France the current model is “2-sessions of physiotherapy-a-week-for-life”[140,141], starting from 3 months of age. **The results of this study should provide high evidence for changing, not only current models of motor rehabilitation in children with CP, but health policies, leading to a new era of early rehabilitation for children with CP.**

- List of references



1. Bleyenheuft Y, Gordon AM. Hand-arm bimanual intensive therapy including lower extremities (HABIT-ILE) for children with cerebral palsy. *Physical & occupational therapy in pediatrics* 2014; **34**(4): 390-403.
2. Bleyenheuft Y, Arnould C, Brandao MB, Bleyenheuft C, Gordon AM. Hand and Arm Bimanual Intensive Therapy Including Lower Extremity (HABIT-ILE) in Children With Unilateral Spastic Cerebral Palsy: A Randomized Trial. *Neurorehabil Neural Repair* 2015; **29**(7): 645-57.
3. Bleyenheuft Y, Ebner-Karestinos D, Surana B, et al. Intensive upper- and lower-extremity training for children with bilateral cerebral palsy: a quasi-randomized trial. *Dev Med Child Neurol* 2017; **59**(6): 625-33.
4. Graham HK, Rosenbaum P, Paneth N, et al. Cerebral palsy. *Nat Rev Dis Primers* 2016; **2**: 15082.
5. Weierink L, Vermeulen RJ, Boyd RN. Brain structure and executive functions in children with cerebral palsy: a systematic review. *Res Dev Disabil* 2013; **34**(5): 1678-88.
6. Krageloh-Mann I, Cans C. Cerebral palsy update. *Brain Dev* 2009; **31**(7): 537-44.
7. Stiles J, Jernigan TL. The basics of brain development. *Neuropsychology review* 2010; **20**(4): 327-48.
8. de Graaf-Peters VB, Hadders-Algra M. Ontogeny of the human central nervous system: what is happening when? *Early human development* 2006; **82**(4): 257-66.
9. Brizzolara D, Pecini C, Brovedani P, Ferretti G, Cipriani P, Cioni G. Timing and type of congenital brain lesion determine different patterns of language lateralization in hemiplegic children. *Neuropsychologia* 2002; **40**(6): 620-32.
10. Chugani HT, Müller R-A, Chugani DC. Functional brain reorganization in children. *Brain and Development* 1996; **18**(5): 347-56.
11. Reid LB, Rose SE, Boyd RN. Rehabilitation and neuroplasticity in children with unilateral cerebral palsy. *Nat Rev Neurol* 2015; **11**(7): 390-400.
12. Bax M, Goldstein M, Rosenbaum P, et al. Proposed definition and classification of cerebral palsy, April 2005. *Dev Med Child Neurol* 2005; **47**(8): 571-6.
13. Novak I, McIntyre S, Morgan C, et al. A systematic review of interventions for children with cerebral palsy: state of the evidence. *Dev Med Child Neurol* 2013; **55**(10): 885-910.
14. Baud O, Daire JL, Dalmaz Y, et al. Gestational hypoxia induces white matter damage in neonatal rats: a new model of periventricular leukomalacia. *Brain pathology (Zurich, Switzerland)* 2004; **14**(1): 1-10.
15. Van Steenwinckel J, Schang AL, Sigaut S, et al. Brain damage of the preterm infant: new insights into the role of inflammation. *Biochemical Society transactions* 2014; **42**(2): 557-63.
16. Favrais G, van de Looij Y, Fleiss B, et al. Systemic inflammation disrupts the developmental program of white matter. *Annals of neurology* 2011; **70**(4): 550-65.
17. Leviton A, Gressens P. Neuronal damage accompanies perinatal white-matter damage. *Trends in neurosciences* 2007; **30**(9): 473-8.
18. Zaehle T, Herrmann CS. Neural synchrony and white matter variations in the human brain--relation between evoked gamma frequency and corpus callosum morphology. *International journal of psychophysiology : official journal of the International Organization of Psychophysiology* 2011; **79**(1): 49-54.
19. Coq JO, Delcour M, Massicotte VS, Baud O, Barbe MF. Prenatal ischemia deteriorates white matter, brain organization, and function: implications for prematurity and cerebral palsy. *Dev Med Child Neurol* 2016; **58 Suppl 4**: 7-11.
20. McKenzie IA, Ohayon D, Li H, et al. Motor skill learning requires active central myelination. *Science* 2014; **346**(6207): 318-22.
21. Xiao L, Ohayon D, McKenzie IA, et al. Rapid production of new oligodendrocytes is required in the earliest stages of motor-skill learning. *Nat Neurosci* 2016; **19**(9): 1210-7.
22. de Faria O, Jr., Pama EAC, Evans K, Luzhynskaya A, Karadottir RT. Neuroglial interactions underpinning myelin plasticity. *Developmental neurobiology* 2017.
23. Xu T, Yu X, Perlik AJ, et al. Rapid formation and selective stabilization of synapses for enduring motor memories. *Nature* 2009; **462**(7275): 915-9.
24. Staudt M, Grodd W, Gerloff C, Erb M, Stitz J, Krageloh-Mann I. Two types of ipsilateral reorganization in congenital hemiparesis: a TMS and fMRI study. *Brain : a journal of neurology* 2002; **125**(Pt 10): 2222-37.
25. Staudt M, Gerloff C, Grodd W, Holthausen H, Niemann G, Krageloh-Mann I. Reorganization in congenital hemiparesis acquired at different gestational ages. *Annals of neurology* 2004; **56**(6): 854-63.
26. Guzzetta A, Bonanni P, Biagi L, et al. Reorganisation of the somatosensory system after early brain damage. *Clinical neurophysiology : official journal of the International Federation of Clinical Neurophysiology* 2007; **118**(5): 1110-21.
27. Li Q, Martin JH. Postnatal development of differential projections from the caudal and rostral motor cortex subregions. *Experimental brain research* 2000; **134**(2): 187-98.
28. Martin JH, Lee SJ. Activity-dependent competition between developing corticospinal terminations. *Neuroreport* 1999; **10**(11): 2277-82.
29. Sampaio-Baptista C, Khrapitchev AA, Foxley S, et al. Motor skill learning induces changes in white matter microstructure and myelination. *J Neurosci* 2013; **33**(50): 19499-503.
30. Demerens C, Stankoff B, Logak M, et al. Induction of myelination in the central nervous system by electrical activity. *Proc Natl Acad Sci U S A* 1996; **93**(18): 9887-92.
31. Gibson EM, Purger D, Mount CW, et al. Neuronal activity promotes oligodendrogenesis and adaptive myelination in the mammalian brain. *Science* 2014; **344**(6183): 1252304.
32. Eyre JA, Taylor JP, Villagra F, Smith M, Miller S. Evidence of activity-dependent withdrawal of corticospinal projections during human development. *Neurology* 2001; **57**(9): 1543-54.
33. Martin JH. The corticospinal system: from development to motor control. *Neuroscientist* 2005; **11**(2): 161-73.

34. Friel K, Chakrabarty S, Kuo HC, Martin J. Using motor behavior during an early critical period to restore skilled limb movement after damage to the corticospinal system during development. *J Neurosci* 2012; **32**(27): 9265-76.
35. Dean DC, 3rd, O'Muircheartaigh J, Dirks H, et al. Mapping an index of the myelin g-ratio in infants using magnetic resonance imaging. *Neuroimage* 2016; **132**: 225-37.
36. Eliasson AC, Nordstrand L, Ek L, et al. The effectiveness of Baby-CIMT in infants younger than 12 months with clinical signs of unilateral-cerebral palsy; an explorative study with randomized design. *Res Dev Disabil* 2018; **72**: 191-201.
37. Eliasson AC, Shaw K, Berg E, Krumlinde-Sundholm L. An ecological approach of Constraint Induced Movement Therapy for 2-3-year-old children: a randomized control trial. *Res Dev Disabil* 2011; **32**(6): 2820-8.
38. Nordstrand L, Holmefur M, Kits A, Eliasson AC. Improvements in bimanual hand function after baby-CIMT in two-year old children with unilateral cerebral palsy: A retrospective study. *Res Dev Disabil* 2015; **41-42**: 86-93.
39. Taub E, Ramey SL, DeLuca S, Echols K. Efficacy of constraint-induced movement therapy for children with cerebral palsy with asymmetric motor impairment. *Pediatrics* 2004; **113**(2): 305-12.
40. DeLuca SC, Case-Smith J, Stevenson R, Ramey SL. Constraint-induced movement therapy (CIMT) for young children with cerebral palsy: effects of therapeutic dosage. *Journal of pediatric rehabilitation medicine* 2012; **5**(2): 133-42.
41. Ferre CL, Brandao MB, Hung YC, Carmel JB, Gordon AM. Feasibility of caregiver-directed home-based hand-arm bimanual intensive training: a brief report. *Developmental neurorehabilitation* 2015; **18**(1): 69-74.
42. Eliasson AC, Nordstrand L, Ek L, et al. The effectiveness of Baby-CIMT in infants younger than 12 months with clinical signs of unilateral-cerebral palsy; an explorative study with randomized design. *Res Dev Disabil* 2017; **72**: 191-201.
43. Can motor skill learning restore impaired corticospinal tract fibers in children with unilateral cerebral palsy? A DTI study. *Developmental Medicine & Child Neurology* 2016; **58**: 42-3.
44. Ronnqvist L, Rosblad B. Kinematic analysis of unimanual reaching and grasping movements in children with hemiplegic cerebral palsy. *Clinical biomechanics (Bristol, Avon)* 2007; **22**(2): 165-75.
45. Jaspers E, Desloovere K, Bruyninckx H, Molenaers G, Klingels K, Feys H. Review of quantitative measurements of upper limb movements in hemiplegic cerebral palsy. *Gait & posture* 2009; **30**(4): 395-404.
46. Sarcher A, Raison M, Leboeuf F, Perrouin-Verbe B, Brochard S, Gross R. Pathological and physiological muscle co-activation during active elbow extension in children with unilateral cerebral palsy. *Clinical neurophysiology : official journal of the International Federation of Clinical Neurophysiology* 2017; **128**(1): 4-13.
47. Gross R, Leboeuf F, Hardouin JB, Perrouin-Verbe B, Brochard S, Remy-Neris O. Does muscle coactivation influence joint excursions during gait in children with and without hemiplegic cerebral palsy? Relationship between muscle coactivation and joint kinematics. *Clinical biomechanics (Bristol, Avon)* 2015; **30**(10): 1088-93.
48. Brochard S, Lempereur M, Mao L, Remy-Neris O. The role of the scapulo-thoracic and gleno-humeral joints in upper-limb motion in children with hemiplegic cerebral palsy. *Clinical biomechanics (Bristol, Avon)* 2012; **27**(7): 652-60.
49. Boyd RN, Ziviani J, Sakzewski L, et al. REACH: study protocol of a randomised trial of rehabilitation very early in congenital hemiplegia. *BMJ open* 2017; **7**(9): e017204.
50. Guzzetta A, Boyd RN, Perez M, et al. UP-BEAT (Upper Limb Baby Early Action-observation Training): protocol of two parallel randomised controlled trials of action-observation training for typically developing infants and infants with asymmetric brain lesions. *BMJ open* 2013; **3**(2).
51. Surveillance of cerebral palsy in Europe: a collaboration of cerebral palsy surveys and registers. Surveillance of Cerebral Palsy in Europe (SCPE). *Dev Med Child Neurol* 2000; **42**(12): 816-24.
52. Roze E, Benders MJ, Kersbergen KJ, et al. Neonatal DTI early after birth predicts motor outcome in preterm infants with periventricular hemorrhagic infarction. *Pediatric research* 2015; **78**(3): 298-303.
53. Bleyenheuft Y, Dricot L, Gilis N, et al. Capturing neuroplastic changes after bimanual intensive rehabilitation in children with unilateral spastic cerebral palsy: A combined DTI, TMS and fMRI pilot study. *Res Dev Disabil* 2015; **43-44**: 136-49.
54. Inguaggiato E, Sgandurra G, Perazza S, Guzzetta A, Cioni G. Brain reorganization following intervention in children with congenital hemiplegia: a systematic review. *Neural plasticity* 2013; **2013**: 356275.
55. Palisano R, Rosenbaum P, Walter S, Russell D, Wood E, Galuppi B. Development and reliability of a system to classify gross motor function in children with cerebral palsy. *Dev Med Child Neurol* 1997; **39**(4): 214-23.
56. Boyd R, Sakzewski L, Ziviani J, et al. INCITE: A randomised trial comparing constraint induced movement therapy and bimanual training in children with congenital hemiplegia. *BMC neurology* 2010; **10**: 4.
57. Greaves S, Imms C, Krumlinde-Sundholm L, Dodd K, Eliasson AC. Bimanual behaviours in children aged 8-18 months: a literature review to select toys that elicit the use of two hands. *Res Dev Disabil* 2012; **33**(1): 240-50.
58. Ferre CL, Brandao M, Surana B, Dew AP, Moreau NG, Gordon AM. Caregiver-directed home-based intensive bimanual training in young children with unilateral spastic cerebral palsy: a randomized trial. *Dev Med Child Neurol* 2017; **59**(5): 497-504.
59. Brandao MB, Mancini MC, Ferre CL, et al. Does Dosage Matter? A Pilot Study of Hand-Arm Bimanual Intensive Training (HABIT) Dose and Dosing Schedule in Children with Unilateral Cerebral Palsy. *Physical & occupational therapy in pediatrics* 2017: 1-16.
60. Sakzewski L, Provan K, Ziviani J, Boyd RN. Comparison of dosage of intensive upper limb therapy for children with unilateral cerebral palsy: how big should the therapy pill be? *Res Dev Disabil* 2015; **37**: 9-16.
61. Van Cauwenberghe E, Gubbels J, De Bourdeaudhuij I, Cardon G. Feasibility and validity of accelerometer measurements to assess physical activity in toddlers. *The international journal of behavioral nutrition and physical activity* 2011; **8**: 67.
62. Krumlinde-sundholm L, Eliasson A-c. Development of the Assisting Hand Assessment: A Rasch-built Measure intended for Children with Unilateral Upper Limb Impairments. *Scandinavian Journal of Occupational Therapy* 2003; **10**(1): 16-26.
63. Greaves S, Imms C, Dodd K, Krumlinde-Sundholm L. Development of the Mini-Assisting Hand Assessment: evidence for content and internal scale validity. *Dev Med Child Neurol* 2013; **55**(11): 1030-7.

64. Holmefur M, Krumlinde-Sundholm L, Eliasson AC. Interrater and intrarater reliability of the Assisting Hand Assessment. *The American journal of occupational therapy : official publication of the American Occupational Therapy Association* 2007; **61**(1): 79-84.
65. Krumlinde-Sundholm L, Holmefur M, Kottorp A, Eliasson AC. The Assisting Hand Assessment: current evidence of validity, reliability, and responsiveness to change. *Dev Med Child Neurol* 2007; **49**(4): 259-64.
66. Vos-Vromans DC, Ketelaar M, Gorter JW. Responsiveness of evaluative measures for children with cerebral palsy: the Gross Motor Function Measure and the Pediatric Evaluation of Disability Inventory. *Disability and rehabilitation* 2005; **27**(20): 1245-52.
67. Russell DJ, Avery LM, Rosenbaum PL, Raina PS, Walter SD, Palisano RJ. Improved scaling of the gross motor function measure for children with cerebral palsy: evidence of reliability and validity. *Physical therapy* 2000; **80**(9): 873-85.
68. Hays RM, McLaughlin JF, Bjornson KF, Stephens K, Roberts TS, Price R. Electrophysiological monitoring during selective dorsal rhizotomy, and spasticity and GMFM performance. *Dev Med Child Neurol* 1998; **40**(4): 233-8.
69. Wei S, Su-Juan W, Yuan-Gui L, Hong Y, Xiu-Juan X, Xiao-Mei S. Reliability and validity of the GMFM-66 in 0- to 3-year-old children with cerebral palsy. *American journal of physical medicine & rehabilitation* 2006; **85**(2): 141-7.
70. Avery LM, Russell DJ, Raina PS, Walter SD, Rosenbaum PL. Rasch analysis of the Gross Motor Function Measure: validating the assumptions of the Rasch model to create an interval-level measure. *Archives of physical medicine and rehabilitation* 2003; **84**(5): 697-705.
71. Hanna SE, Bartlett DJ, Rivard LM, Russell DJ. Reference curves for the Gross Motor Function Measure: percentiles for clinical description and tracking over time among children with cerebral palsy. *Physical therapy* 2008; **88**(5): 596-607.

Proposal Template
