# Supplementary material for: Changes Induced by Early Hand-Arm Bimanual Intensive Therapy Including Lower Extremities in Young Children With Unilateral Cerebral Palsy: A Randomized Clinical Trial
Source: JAMA Pediatr. 2023 Nov 6;178(1):19–28. doi: 10.1001/jamapediatrics.2023.4809 (PMC10628844; doi:10.1001/jamapediatrics.2023.4809)
Supplement: Supplement 3. — Data sharing statement [file jamapediatr-e234809-s003.pdf]

Data Sharing Statement

Araneda. Changes Induced by Early Hand-Arm Bimanual Intensive Therapy Including Lower Extremities in Young Children With Unilateral Cerebral Palsy. *JAMA Pediatr*. Published November 06, 2023. doi:10.1001/jamapediatrics.2023.4809

Data

Data available: Yes
Data types: Deidentified participant data
How to access data: The data that support the findings of this study will be available upon reasonable request from the corresponding author (yannick.bleyenheuft@uclouvain.be)
When available: With publication

Supporting Documents

Document types: Informed consent form
How to access documents: The data that support the findings of this study will be available upon reasonable request from the corresponding author (yannick.bleyenheuft@uclouvain.be)
When available: With publication

Additional Information

Who can access the data: Researchers whose proposed use of the data will be considered appropiated upon request
Types of analyses: for any purpose
Mechanisms of data availability: after approval of a proposal
